# RETRACTED: Muhammad et al. Hesperetin, a Citrus Flavonoid, Attenuates LPS-Induced Neuroinflammation, Apoptosis and Memory Impairments by Modulating TLR4/NF-κB Signaling. *Nutrients* 2019, *11*, 648

**DOI:** 10.3390/nu18030415

**Published:** 2026-01-27

**Authors:** Tahir Muhammad, Muhammad Ikram, Rahat Ullah, Shafiq Ur Rehman, Myeong Ok Kim

**Affiliations:** Division of Applied Life Science (BK 21), College of Natural Sciences, Gyeongsang National University, Jinju 52828, Republic of Korea; mtahir.khan@gnu.ac.kr (T.M.); qazafi417@gnu.ac.kr (M.I.); rahatullah1414@gnu.ac.kr (R.U.); shafiq12@gnu.ac.kr (S.U.R.)

The journal retracts the article “Hesperetin, a Citrus Flavonoid, Attenuates LPS-Induced Neuroinflammation, Apoptosis and Memory Impairments by Modulating TLR4/NF-κB Signaling” [1] cited above.

Following publication, concerns were brought to the attention of the Editorial Office regarding the integrity of a number of images presented in this publication [1].

Adhering to our standard procedure, an investigation was conducted by the Editorial Office and Editorial Board that identified indications of inappropriate editing and partial duplication between Figures 2A, 4B, and 7B presented in this article [1]. While the authors collaborated within this process, raw material meeting the journals requirements for original images (https://www.mdpi.com/journal/nutrients/instructions#oriimages, accessed on 18 November 2025) could not be provided for Editorial Board evaluation. Consequently, the Editorial Board has lost confidence in the reliability of the findings and has decided to retract this publication, as per MDPI’s retraction policy (https://www.mdpi.com/ethics#_bookmark30, accessed on 18 November 2025).

This retraction was approved by the Editor-in-Chief of *Nutrients*.

The authors did not agree to this retraction.
